# Association between the atherogenic index of plasma and abdominal aortic calcification in adults: a cross-sectional study

**DOI:** 10.1186/s12889-024-19862-3

**Published:** 2024-09-06

**Authors:** Cong Xu, Shuwan Xu, Peibiao Mai, Jiao Tang, Jiahua Xu, Huanji Zhang

**Affiliations:** 1 Department of Cardiology, The Eighth Affiliated Hospital of Sun Yat-Sen University, Sun Yat-Sen University, No. 3025 Shennan Middle Road, Shenzhen, Guangdong People’s Republic of China; 2 Guangdong Innovative Engineering and Technology Research Center for Assisted Circulation, Sun Yat-sen University, Shenzhen, 518033 People’s Republic of China

**Keywords:** Abdominal aortic calcification, Cross-sectional study, Atherogenic index of plasma, NHANES, Insulin resistance

## Abstract

**Background:**

Atherogenic index of plasma (AIP) index is an important marker of insulin resistance and a significant risk factor for cardiovascular disease. Abdominal aortic calcification (AAC) is significantly associated with subclinical atherosclerotic disease. However, there are no studies that have examined the relationship between AIP index and AAC, so we investigated the potential association between them in the general population.

**Methods:**

This was a cross-sectional study using data from the National Health and Nutrition Examination Survey (NHANES, 2013–2014). The association of AIP with AAC was estimated by multivariable regression analysis.

**Results:**

After adjusting for confounders, the odds of extensive AAC doubled per unit increase in the AIP index (OR = 2.00, 95% CI: 1.05, 3.83; *P* = 0.035). The multivariable OR and 95% CI of the highest AIP index tertile compared with the lowest tertile was significantly different. (OR = 1.73, 95% CI: 1.05, 2.83; *P* = 0.031). The subgroup analyses indicated that the association was consistent irrespective of age, sex, hypertension, diabetes, smoking status, eGFR and hypercholesteremia.

**Conclusions:**

The AIP index was independently associated with the presence of extensive AAC in the study population. Further studies are required to confirm this relationship.

**Supplementary Information:**

The online version contains supplementary material available at 10.1186/s12889-024-19862-3.

## Introduction

Cardiovascular disease (CVD) persists as the leading cause of mortality in both the United States and globally. The age-standardized CVD mortality rate varied by region, from 73.6 per 100,000 in high-income Asia-Pacific to 432.3 per 100,000 in Eastern Europe in 2022. Early detection of atherosclerosis at the subclinical stage was pivotal for timely prevention and intervention of CVD [1]. Vascular calcification (VC) was defined as the deposition of minerals in the form of calcium-phosphate complexes in the vascular system. Traditionally, calcification had been divided into two forms, depending on where the minerals were deposited, intimal calcification and medial calcification [2]. Abdominal aortic calcification (AAC) was a common type of vascular calcification (VC) [3]. The prevalence of AAC increased with age, from 60% at 65–69 years to 96% at 85 years and older [4]. In addition to being a stable marker of atherosclerotic vascular disease, it had been found to predict cardiovascular events and all-cause mortality [5–8]. Several epidemiologic studies had shown that the severity of AAC assessed on spinal radiographs is a useful predictor of CVD morbidity and mortality [8–12]. The predictive capacity of AAC for CVD, independent of coronary artery calcification (CAC), with its correlation to total mortality surpassing that of CAC, was well documented [13].

The plasma atherogenic index (AIP), which was the log-transformed ratio of TG to HDL-C molar concentration, had emerged as a novel marker of atherosclerosis and CVD [14–16]. Elevated TG and low HDL levels were strong markers of CVD, and elevated TG levels led to increased levels of small dense LDL, ultimately increasing CV risk [17]. AIP was an important predictor of atherosclerosis and cardiovascular disease, and was superior to standard atherogenic lipid profiles [16, 17]. For example, in a large cohort study of postmenopausal women undergoing coronary angiography, Guo et al. demonstrated in univariate and multivariate regression analyses that AIP was superior to traditional lipid markers in predicting coronary artery disease [18].

However, to the best of our knowledge, the relationship between AIP and AAC had not been previously studied; therefore, we aimed to investigate the association between AIP and AAC in the US population using the 2013–2014 National Health and Nutrition Examination Survey (NHANES). We hypothesized that AIP levels would be positively associated with severe AAC.

## Methods

### Data sources

The NHANES was a continuous survey that selects a group of representative American people by means of complex and multistage probability sampling and aimed to evaluate the health and nutrition status of American adults and children [19]. The data used in this study were all from the 2013–2014 National Health and Nutrition Examination Survey (NHANES) database. The Ethics Review Committee of the National Center for Health Statistics (NCHS) approved the NHANES research plan. All the research participants provided written informed consent.

### Independent and dependent variables

Blood collection was performed in the morning after fasting to collect high-density lipoprotein (HDL) and triglycerides. The AIP index was calculated as follows: AIP = Log_10_ [triglycerides (mmol/L)/high-density lipoprotein cholesterol (mmol/L)] [20].

In this study, abdominal aortic calcification (AAC) was obtained by dual energy X-ray absorptiometry (DXA). It had been shown that the images obtained by this method have fairly good sensitivity and specificity [21, 22]. Data collection and scan analysis were tightly controlled for quality, with participant and phantom scans evaluated by UCSF using standard and NHANES-specific radiology protocols. Images were read by a single reader at the UCSF quality control center, who was trained by Dr. John Schousboe. More detailed information can be found on the official NHANES website at DXXAAC_H (cdc.gov). AAC-24 method for the assessment of abdominal aortic calcification [23, 24]. In the AAC-24 scoring system, the anterior and posterior aortic walls was divided into eight segments aligned with the L1-L4 vertebrae. Aortic calcification in each segment was visually graded: “0” for none, “1” for less than one-third calcified, “2” for one-third to two-thirds calcified, and “3” for more than two-thirds. The anterior and posterior walls were scored separately, with a per-segment range of “0–3” and a total possible score of “0–24”. Reference to relevant studies, a Kauppila score 5 was used to define extensive AAC [25, 26]. Participants ultimately included in the study were divided into two groups “Without extensive AAC (AAC score<5)” and “With extensive AAC (AAC score ≥ 5)”.

### Other variables

Other covariates, including demographics, comorbidities, lifestyle variables, body mass index (BMI), uric acid, estimated glomerular filtration rate (eGFR), serum markers of bone mineral metabolism were selected. All information were obtained through standard questionnaires and MEC.

The race variable included Mexican American, other Hispanic, non-Hispanic White, non-Hispanic Black, and other races. BMI (kg/m^2^) was calculated as weight in kilograms divided by the square of height in meters. Poverty was defined as having a family income: poverty ratio ≤ 1.3 [27]. Hypertension was defined as self-reported physician-diagnosed hypertension, taking antihypertensive medications, or blood pressure measurement ≥ 140/90mmHg [28]. Diabetes mellitus was defined as self-reported physician-diagnosed diabetes, taking oral hypoglycemic agents or insulin, a fasting glucose level ≥ 126 mg/dL, or a plasma glucose level ≥ 200 mg/dL 2 h after oral glucose tolerance test [29]. High cholesterol level was defined as a total cholesterol level ≥ 240 mg/dL or the use of medication for hypercholesterolemia [30]. Smokers were defined as those who had smoked at least 100 cigarettes in their lifetime. The eGFR was calculated using the Chronic Kidney Disease Epidemiology Collaboration creatinine equation [31]. Serum markers of bone mineral metabolism included total 25-hydroxyvitamin D, serum calcium and phosphorus [32].

### Statistical analysis

In our study, continuous variables were presented as means and standard deviations, and categorical variables were presented as numbers (n) and percentages (%). We tested differences in characteristics between groups (with extensive AAC and without extensive AAC) using Student’s *t*-tests for continuous variables and Chi-square test for categorical variables. We assessed the correlation between different AIP index as continuous variables and AAC using both univariate and multivariate logistic regression model, expressing the relationship with OR values and 95% confidence intervals (95% CI). In addition to the unadjusted model, potential covariates were progressively adjusted in three models. Model 1 was adjusted for age, gender, and race. Model 2 was additionally adjusted for BMI, hypertension, diabetes mellitus, high cholesterol, smoking status, education and poverty. Model 3 was further adjusted for uric acid, total 25-hydroxyvitamin D, calcium, phosphorus, eGFR. Based on the results of these analyses, We further assessed the differences in AAC risk between the different AIP tertile groups (using the Tertile 1 group as a reference) by equating the AIP index into 3 groups, Tertile 1 (−1.09 to −0.23), Tertile 2 (−0.23 to 0.05), and Tertile 3 (0.05 to 1.00). Tests for linear trends across the AIP index categories were conducted using an independent ordinal variable in all models. In addition, we used restricted cubic spline (RCS) curves based on Model 3 to explore any non-linear relationship between AIP index and AAC, and generalized linear models on AIP index and AAC were used to further enhance the stability of the results.

To explore whether the association between the AIP index and extensive AAC was modified by age, sex, smoking status, and comorbidities, we performed subgroup analyses by age group (<60 or 60 years), sex (male or female), hypertension (yes or no), diabetes (yes or no), high cholesterol (yes or no) and smoking (yes or no), and examined the interactions between the stratifying variables and the AIP index.

All statistical analyses were performed using IBM SPSS Statistics 21 (IBM SPSS, Turkey) program and R version 3.6.1 (R Foundation for Statistical Computing, Vienna, Austria). P-value < 0.05 was considered significant.

## Results

**Fig. 1:**
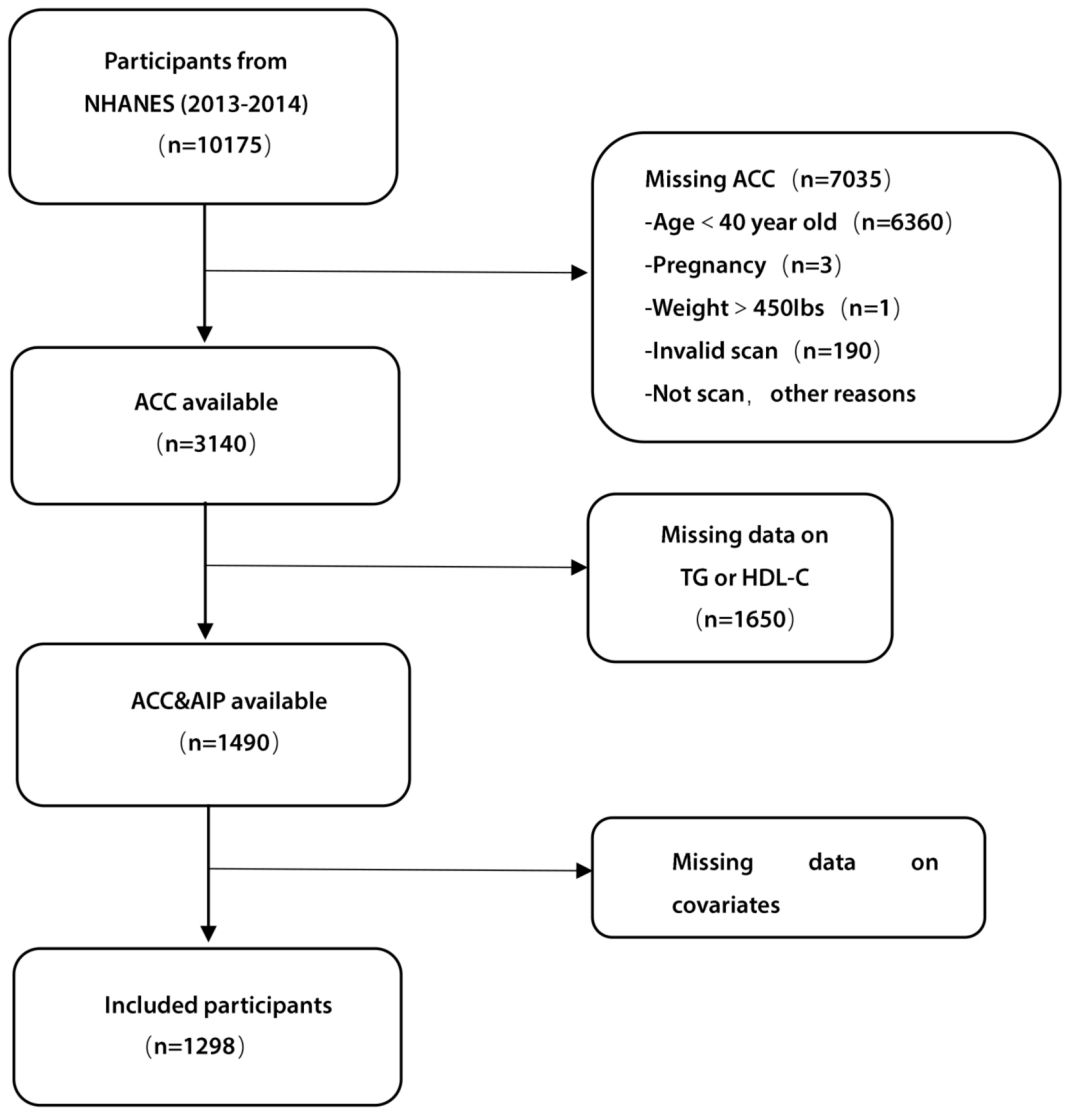
Flow chart of participants’ selection

In the NHANES 2013–2014 cohort, a total of 10,175 participants completed interviews. This included 3140 participants with valid data on AAC scores. We excluded participants with missing data on triglycerides or high-density lipoprotein (HDL) (*n* = 1650) or other covariates (*n* = 192), such as demographics, co-morbidities, and blood biochemistry indicators. Finally, a total of 1298 participants were included in this cross-sectional study. A detailed flow chart describing participant selection is shown in Fig. 1.

### Baseline characteristics of study participants

Table 1 present the baseline characteristics of the two groups. Compared with those without extensive AAC, participants with extensive AAC were older and more likely to be black, female, and smokers. These participants also had a higher prevalence of hypertension, diabetes, high cholesterol levels, higher levels of uric acid, 25-hydroxyvitamin D, and lower eGFR.

**Table 1:** Characteristics of the study participants

Characteristics	All (*N* = 1298)	Without extensive AAC (*N* = 1112)	With extensive AAC (*N* = 186)	*p*-value
Age (years)	59.0 ± 12.3	57.2 ± 11.6	69.8 ± 10.3	<0.001
Male	629 (48.5%)	540 (48.6%)	89 (47.8%)	0.857
Race				0.001
Mexican American	161 (12.4%)	145 (13.0%)	16 (8.6%)	
Non-Hispanic White	113 (8.7%)	104 (9.4%)	9 (4.8%)	
Non-Hispanic Black	605 (46.6%)	492 (44.2%)	113 (60.8%)	
Other Hispanic	242 (18.6%)	211 (19.0%)	31 (16.7%)	
Other Race	177 (13.6%)	160 (14.4%)	17 (9.1%)	
BMI (kg/m^2^)	28.3 ± 5.6	28.5 ± 5.8	27.0 ± 4.3	<0.001
SBP (mmHg)	126.3 ± 18.9	125.0 ± 18.5	133.8 ± 19.8	<0.001
DBP (mmHg)	68.6 ± 13.7	69.4 ± 12.9	63.6 ± 17.2	<0.001
Hypertension	555 (42.8%)	427 (38.4%)	128 (68.8%)	<0.001
Diabetes mellitus	534 (41.1%)	437 (39.3%)	97 (52.2%)	0.001
High cholesterol	490 (37.8%)	381 (34.3%)	109 (58.6%)	<0.001
Poverty income ratio	385 (29.7%)	323 (29.0%)	62 (33.3%)	0.236
Less than high school diploma	561 (43.2%)	464 (41.7%)	97 (52.2%)	0.008
Smoker	606 (46.7%)	494 (44.4%)	112 (60.2%)	<0.001
TC (mmol/L)	5.0 ± 1.1	5.0 ± 1.1	4.8 ± 1.1	0.017
LDL-C (mmol/L)	3.0 ± 0.9	3.0 ± 0.9	2.8 ± 1.0	0.001
HDL-C (mmol/L)	1.4 ± 0.4	1.4 ± 0.4	1.4 ± 0.5	0.729
TG (mmol/L)	1.3 ± 0.7	1.3 ± 0.7	1.4 ± 0.7	0.071
FPG (mmol/L)	6.6 ± 0.4	6.6 ± 0.4	6.7 ± 0.5	0.167
Serum uric acid (µmol/L)	326.1 ± 82.9	322.8 ± 80.9	345.8 ± 91.6	<0.001
Total 25-hydroxyvitamin D (nmol/L)	70.9 ± 28.7	69.7 ± 28.1	77.9 ± 31.2	0.001
Serum calcium (mmol/L)	2.4 ± 0.1	2.4 ± 0.1	2.4 ± 0.1	0.081
Serum phosphorus (mmol/L)	1.2 ± 0.2	1.2 ± 0.2	1.2 ± 0.2	0.016
eGFR (ml/min/1.73m^2^)	86.3 ± 21.9	88.9 ± 20.5	70.9 ± 23.8	<0.001

Values are presented as mean ± standard deviation or n (%)

*Abbreviations AAC* abdominal aortic calcification, *BMI* body mass index, *SBP* systolic blood pressure, *DBP* diastolic blood pressure, *TC* total cholesterol, *LDL-C* low-density lipoprotein cholesterol, *HDL-C* high-density lipoprotein cholesterol, *TG* triglycerides, *FPG* fasting plasma glucose, *eGFR* estimated glomerular filtration rate

### The association between AIP index and extensive AAC

Table 2 showed the relationship between extensive AAC and the AIP index as continuous and categorical variables. When the AIP index was analyzed as a continuous variable, per unit increase in the AIP index resulted in higher odds of extensive AAC in the univariate logistic regression model (OR = 1.58, 95% CI: 0.97, 2.60), but present marginal significance (*p* = 0.068). The association were statistically significant in all multivariate logistic regression models after adjusting for several covariates including age, sex, race, BMI, hypertension, diabetes mellitus, high cholesterol, smoking status, education, poverty, uric acid, total 25-hydroxyvitamin D, calcium, phosphorus and eGFR. (Model 1: OR = 2.32, 95% CI: 1.30, 4.12; Model 2: OR = 2.33, 95% CI: 1.24, 4.40; Model 3: OR = 2.00, 95% CI: 1.05, 3.83). When the AIP index was treated as a categorical variable based on tertiles, compared with participants in the lowest tertile, those in the highest tertile of the AIP index had higher odds for extensive AAC in all four models (unadjusted model: OR = 1.56; 95% CI: 1.06, 2.30; Model 1: OR = 1.91, 95% CI: 1.24, 2.95; Model 2: OR = 1.89, 95% CI: 1.17, 3.06; Model 3: OR = 1.73, 95% CI: 1.05, 2.83; all p for trend < 0.05). In the generalized linear model, the risk of AAC increased by 39% for each unit increase in the AIP index after adjusting for relevant variables (Table 3 in Supplement 1).

**Table 2:** Multivariable-adjusted ORs and 95% confidence intervals of the AIP index associated with extensive AAC

AIP index	Unadjusted	Model 1^a^	Model 2^b^	Model 3^c^
	OR (95%CI) *p*-value	OR (95%CI) *p*-value	OR (95%CI) *p*-value	OR (95%CI) *p*-value
Per unit increase	1.58 (0.97, 2.60) 0.068	2.32 (1.30, 4.12) 0.004	2.33 (1.24, 4.40) 0.009	2.00 (1.05, 3.83) 0.035
**Categorical**				
Tertile1	1.0	1.0	1.0	1.0
Tertile2	1.30 (0.88, 1.94) 0.191	1.43 (0.92, 2.20) 0.110	1.53 (0.97, 2.42) 0.070	1.53 (0.96, 2.44) *p* = 0.077
Tertile3	1.56 (1.06, 2.30) 0.025	1.91 (1.24, 2.95) 0.003	1.89 (1.17, 3.06) 0.009	1.73 (1.05, 2.83) 0.031
P for trend	0.025	0.003	0.010	0.035

^a^Model 1 adjusted for age, gender and race

^b^Model 2 further adjusted for BMI, hypertension, diabetes mellitus, high cholesterol, smoking status, education and poverty

^c^Model 3 further adjusted for uric acid, total 25-hydroxyvitamin D, calcium, phosphorus, eGFR

**Fig. 2:**
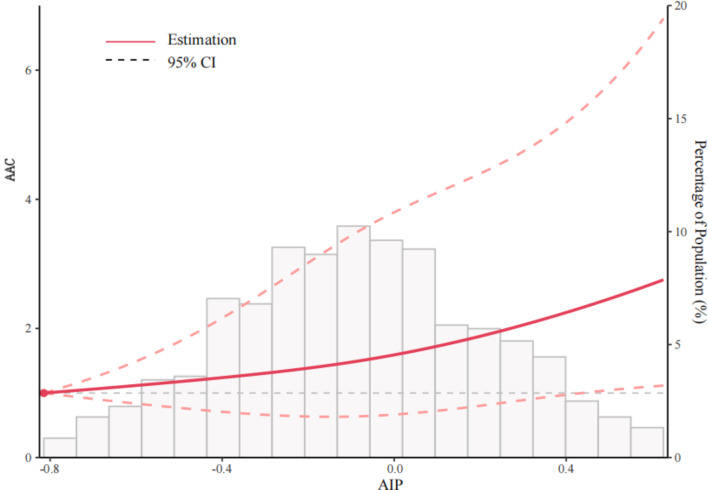
Restricted cubic spline fitting for the association between AIP with AAC

**Fig. 3:**
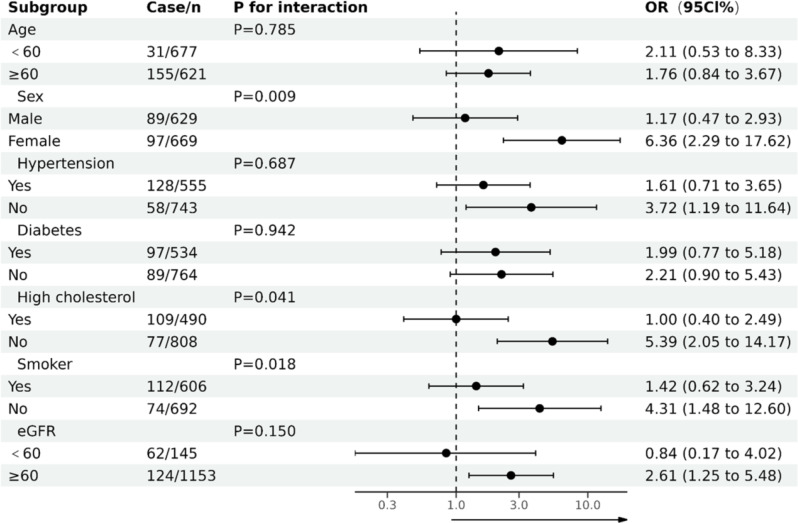
Stratified associations between AIP index and AAC according to baseline characteristics

As seen in Fig. 2, the dose-response analysis with a restricted cubic spline model showed a nearly linear relationship between the AIP index and the odds of extensive AAC after adjustment for multiple potential covariates in Model 3 (Table 2) (p for nonlinear = 0.741). The subgroup analyses treating the AIP index as a continuous variable (per unit increase) were shown in Fig. 3. All associations were positive in the different subgroups. Significant interactions were found between the AIP index and sex (p for interaction = 0.009), high cholesterol status (p for interaction = 0.041) and smoking status (p for interaction = 0.018).

## Discussion

In this cross-sectional study, we explored the relationship between different AIP levels and AAC in the general US population. Our findings revealed that higher AIP indices showed a near-linear response relationship with increased odds of extensive AAC, even after adjusting for multiple potential covariates including demographics, cardiovascular risk factors, and more. Stratified analyses indicated that the direction of the relationship between AIP and extensive AAC was approximately consistent across different subgroups, mirroring the overall trend observed in the aggregate population. The generalized linear model on AIP index and abdominal aorta calcification further enhanced the stability of the results.

Arterial calcification increased the burden of atherosclerosis and atherosclerosis and emerged as an important risk factor for cardiovascular disease [33]. Although there were no studies reporting the relationship between AIP index and AAC, some studies had explored the relationship between AIP and CAC progression. For example, Nam et al. reported in a longitudinal study that among Korean subjects without coronary artery disease, those with higher AIP had an increased risk of cardiovascular disease and higher CACS and were more likely to have CAC progression within 4 years [34]. Won et al. found that AIP was independently associated with CAC progression, especially in the absence of severe CAC (CACSs > 100) at baseline [35]. In the present study, we found that higher AIP index was not significantly associated with severe abdominal aortic calcification in the unadjusted model, whereas this association became significant after adjusting for relevant cardiovascular risk factors, and the implications of this finding for clinical practice were far-reaching. High AIP index may be an independent predictor of abdominal aortic calcification, but its predictive ability may be affected by other factors. The development of abdominal aortic calcification involved multiple pathological processes, including inflammation, oxidative stress, and metabolic disorders [2], and disorders such as hypertension and diabetes mellitus may also affect the development and progression of abdominal aortic calcification, and the AIP index reflected the levels of inflammation and oxidative stress, whereas it may be a consequence or intermediate phenotype of these coexisting diseases. However, the specific role of the AIP index in these processes still required further investigation. Therefore, in actual clinical diagnosis and prognostic assessment, physicians need to consider multiple factors simultaneously, including controlling risk factors such as blood pressure, blood glucose, and lipids, not just the AIP index.

Based on the available literature, we speculated on the following potential mechanisms for the association between the AIP index and abdominal aortic calcification. Firstly, one possible mechanism could involve the insulin resistance (IR). A cross-sectional study found that the TG/HDL ratio was positively associated with IR in a nonlinear reciprocal pattern in the U.S. population [36]. The AIP index was a relatively convenient biomarker of IR because the cost of routine measurements of both triglycerides and HDL cholesterol was lower than insulin-based markers in clinical practice. High TG/HDL ratios would lead to decreased retention of fatty acids, resulting in more fatty acids being translocated to the liver for TG synthesis [37], TG-rich lipoproteins accelerated the synthesis of factors such as leptin, angiotensinogen, tumor necrosis factor α, interleukin 6, fibrinogen activator inhibitor 1, transforming growth factor B, adiponectin, lipocalin. Related research suggested these factors were risk factors for insulin resistance or diabetes [38, 39]. Moreover, IR had been shown to be associated with systemic cardiovascular risk factors, including hyperglycemia, hypertension, and hyperlipidemia. A retrospective observational study found that the metabolic syndrome, which consisted of cardiometabolic risk factors such as HDL, was associated with AAC in U.S. adults [40]. In addition, IR may play a role in atherogenesis and advanced plaque progression, with defected in insulin signaling pathways in atherosclerotic lesion cells [41]. However, the interplay between IR and AAC had not been extensively probed. A multiethnic cross-sectional study suggested a significant association between IR and the presence of AAC in nondiabetic individuals, although this link attenuated when adjusted for other variables like abdominal muscle mass, subcutaneous fat area, and visceral fat area [42]. Therefore, more relevant studies are needed to explore the potential mechanisms of IR action in AAC.

Secondly, another potential mechanism may involve dysregulation of lipid metabolism. The AIP index was strongly associated with dyslipidemia. Elevated levels of AIP index tended to be associated with elevated triglyceride levels and decreased high-density lipoprotein (HDL) cholesterol, which was a known risk factor for atherosclerosis and calcification. Dyslipidemia could lead to endothelial dysfunction, oxidative stress, and inflammation [43]. Moreover, AIP index had been shown to have a proinflammatory effect and may contribute to the activation of inflammatory cells and the release of cytokines and chemokines, which further exacerbated the inflammatory response in the vascular wall and promote calcification [44]. All of these were key processes involved in the development of vascular calcification, including calcification of the abdominal aorta [2].

Thirdly, Body mass index (BMI), a measure of obesity, may also play a potential role in the relationship between AIP index and abdominal aortic calcification. Obesity was a chronic low-grade inflammatory state, which further exacerbated the inflammatory response induced by dyslipidemia by increasing circulating inflammatory factors, such as TNF-α and interleukin 6 (IL-6) [45]. Adipose tissue not only served as a site for energy storage, but also secreted a variety of adipokines, which played an important role in the regulation of lipid metabolism and the inflammatory response role [46]. In addition, obesity was associated with elevated triglyceride levels and reduced high-density lipoprotein (HDL) cholesterol levels [47]. This led to elevated AIP index and promotion of calcification. In conclusion, although the specific mechanisms by which a higher AIP index were associated with severe abdominal aortic calcification were not fully understood, the mechanisms described above were plausible avenues that warranted further investigation. Future studies should aim to elucidate these mechanisms in more detail to better understand the underlying pathophysiology and to develop targeted interventions for the management of AIP index and the prevention and treatment of abdominal aortic calcification.

In stratified analyses, the correlation between AIP index and AAC showed a trend relationship consistent with the primary results. However, we found that in some subgroups such as participants less than 60 years old, men, smokers, hypertensive, diabetic, and hypercholesterolemic participants, the ORs were lower than the corresponding subgroups and did not reach statistical significance. Hypertension and smoking were clear risk factors for cardiovascular disease and can significantly increase the risk of cardiovascular disease when combined with insulin resistance or dyslipidemia [48–50], but a prospective cohort study found that among 3777 individuals aged > 30 years without a history of cardiovascular disease and who were not using antidiabetic medications (median follow-up period > 10 years), higher HOMA-IR was significantly associated with a higher risk of cardiovascular disease in the group without hypertension, but no significant association was found in the hypertensive group, and the presence of hypertension altered the effect of insulin resistance on CVD occurrence [51]. However, no other study had yet found whether smoking, poorer renal function, etc., weakened the association between the AIP index and cardiovascular disease. Therefore, the relationship between the AIP index and abdominal aortic calcification in specific populations had not been fully clarified, and further studies are needed to be investigate. In addition, only 31 and 58 participants with extensive AAC were included in the stratified subgroups of age < 60 years and no hypertension, respectively. Potential bias may have resulted from the small sample size, and the results need to be validated in a larger, specific population in the future.

This was the first study to explore the association between the AIP Index and broad AAC in a nationally representative sample of adults in the United States. Our study had a rigorous research protocol and quality control. However, this study had some limitations. First, as a cross-sectional observational study, it was not possible to demonstrate whether there was a causal relationship between the AIP index and extensive AAC. Second, only adults > 40 years of age were included in this study, which could be a potential source of bias and thus further broader population exploration is needed. Third, because all population participants were U.S. residents and this study did not consider pregnant women, children, or individuals with specific medical conditions, it was difficult to know whether the results of this study would be applicable to these populations.

## Conclusion

In a nationally representative sample of adults in the United States, the AIP index was independently associated with a wide range of AACs determined using DXA. In addition, there was a significant positive correlation between AIP and extensive ACC in low-risk groups such as the female population, nonsmokers, those without hypertension, those without high cholesterol, and those with high glomerular filtration rates. Our findings may provide support for further large-scale prospective studies to elucidate the exact causality of this relationship.

## Electronic supplementary material

Below is the link to the electronic supplementary material.

Supplementary Material 1

## Data Availability

The data underlying this article will be shared upon reasonable request to the corresponding author.
