# Supplementary material for: Association between the atherogenic index of plasma and abdominal aortic calcification in adults: a cross-sectional study
Source: BMC Public Health. 2024 Sep 6;24:2431. doi: 10.1186/s12889-024-19862-3 (PMC11380399; doi:10.1186/s12889-024-19862-3)
Supplement: Supplementary file 1 — Supplementary Material 1 [file 12889_2024_19862_MOESM1_ESM.docx]

**Supplement 1**

Association between the atherogenic index of plasma and abdominal

aortic calcification in adults: A cross-sectional study

Cong Xu^1,*^ , Shuwan Xu^1,*^ , Peibiao Mai^1^ , Jiao Tang^1^ , Jiahua Xu^1^ , Huanji Zhang^1, 2^

^1^Cardiovascular Department, the Eighth Affiliated Hospital of Sun Yat-Sen University, Sun Yat-Sen University, Shenzhen, Guangdong, People’s Republic of China

^2^Guangdong Innovative Engineering and Technology Research Center for Assisted Circulation, Sun Yat-sen University, Shenzhen 518033, China

*These authors contributed equally to this work


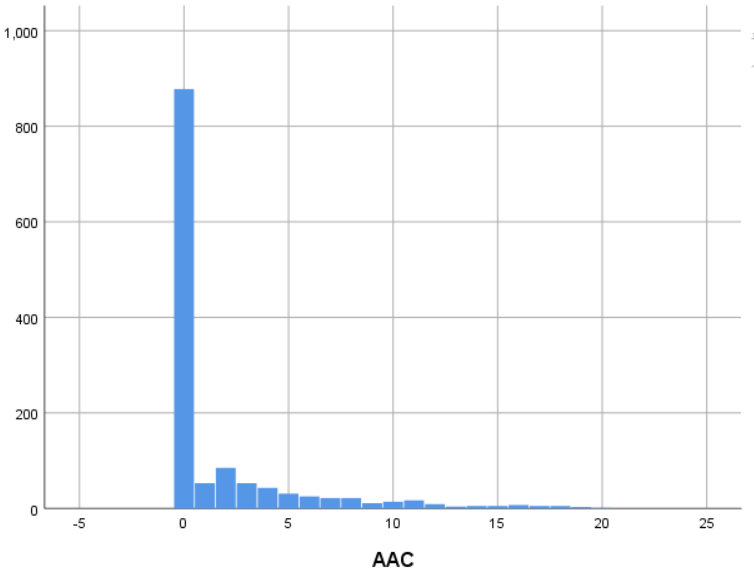


**Figure 4** Distribution histogram of AAC

| **Table 3** Relationship between AIP index and AAC in a generalized linear model | | | | |
| --- | --- | --- | --- | --- |
| **AIP index** | **Unadjusted** | **Model1** | **Model2** | **Model3** |
|  | **OR（95%CI）**  **p-value** | **OR（95%CI）**  **p-value** | **OR（95%CI）**  **p-value** | **OR（95%CI）**  **p-value** |
| per unit increase | 1.27（1.11,  1.44）＜0.01 | 1.47（1.29,  1.69）＜0.01 | 1.49（1.29,  1.73）＜0.01 | 1.39（1.20,  1.61）＜0.01 |
| a Model 1 adjusted for age, gender and race.  b Model 2 further adjusted for BMI, hypertension, diabetes mellitus, high cholesterol, smoking status, education and poverty.  c Model 3 further adjusted for uric acid, total 25-hydroxyvitamin D, calcium, phosphorus, eGFR. | | | | |
